# We Are Very Similar but Not Really: The Moderating Role of Cultural Identification for Refugee Resettlement of Venezuelans in Colombia

**DOI:** 10.3389/fpsyg.2020.569394

**Published:** 2020-11-24

**Authors:** Yarid Ayala, Jaime Andrés Bayona, Aysegul Karaeminogullari, Jesús Perdomo-Ortíz, Mónica Ramos-Mejía

**Affiliations:** ^1^Department of Management and Leadership, Tecnologico de Monterrey, School of Business, Mexico City, Mexico; ^2^Business Department, School of Economics and Business, Pontificia Universidad Javeriana, Bogotá, Colombia

**Keywords:** career adaptability, social netwoks, cultural identification, refugees, resettlement sucess

## Abstract

This study aims to test the theoretical model of career adaptability of refugees to investigate the dynamics of successful resettlement. The theoretical model is grounded on career construction and social network theory. We employ quantitative and qualitative methodologies to test the model in a sample of Venezuelans living and working in Colombia. The quantitative results provide partial support for Campion’s model. However, we test an alternative model and find that career adaptability has a direct relationship with subjective resettlement (i.e., life satisfaction and psychological health). In addition, cultural identification plays a buffering role on the harmful effects of discrimination on subjective resettlement. Qualitative results from eight in-depth interviews shed light on the process of refugee resettlement, thus revealing the role of social networks. Our study contributes to previous research on refugees by testing, adapting, and expanding a novel model of work resettlement and focusing on a group of refugees transitioning from one emerging country to another emerging country.

## Introduction

Currently, an estimated 70.8 million people have been forcibly displaced worldwide (United Nations High Commissioner for Refugee, 2019). In a recent report, the UN declared that given the deterioration of the socioeconomic and political situation, Venezuela is one of the major countries in the Americas experiencing dramatic displacement. Over 1.5 million people have left Venezuela to settle in neighboring countries, and thousands of them remain in an unsteady economic situation, making them particularly vulnerable to exploitation, trafficking, violence, forced recruitment, sexual abuse, discrimination, xenophobia, and other social malaises (United Nations High Commissioner for Refugee, 2017). Currently, Colombia is one of the major host countries in the ongoing exodus of hundreds of thousands of Venezuelans. According to the National Office of Migration in Colombia, more than 1,260,000 Venezuelans are living in Colombia with the majority being located in the capital city of Bogotá (Migración Colombia, 2019).

Many previous studies focused on refugees living in various countries, such as Austria, Germany, Turkey, United States, Greece, or the Netherlands (Baranik et al., 2018; Eggenhofer-Rehart et al., 2018; Gericke et al., 2018; Knappert et al., 2018; Pajic et al., 2018; Wehrle et al., 2018). Such studies not only identified obstacles such as hostile labor markets, social rejection, exploitation legitimation, or long-term unemployment but also suggested coping mechanisms, such as social capital, psychological capital, reflection–relaxation, or problem-solving, that accordingly hinder or assist refugees in navigating their careers and overcoming the associated work-related challenges. Another stream of studies examined expatriates to shed light on the dynamics of cross-cultural adaptation (Chen et al., 2019; Giorgi et al., 2020). However, little is known about the resettlement of refugees to a country that shares a common cultural heritage. What barriers do they face? How do they cope with them? Do they depend on specific strategies? What factors significantly help them to improve their fit in their new environments in terms of objective and subjective resettlement success factors? Examining these questions in the case of Venezuelans who have settled in Colombia is distinctive from the vast majority of existing refugee studies given that our study addresses displacement across countries that share a common cultural heritage (e.g., both countries speak the same language and have strong historical and cultural communalities).

In contrast, previous research presented less apparent similarities between the home and host countries. Since the 1960, Venezuela has been home to many Colombians, thus offering a chance to work and live in peace. Furthermore, the oil-rich country was constantly better off in terms of economy compared with Colombia, which implies that Venezuelan refugees are leaving a previously wealthy country to settle into a developing country, which is in an on-going process of peace recovery. This distinctiveness renders the investigation on the case of Venezuelans notable because it presents a unique example of migration where refugees may have high cultural identification with the host country.

## Hypotheses

**Figure 1:**
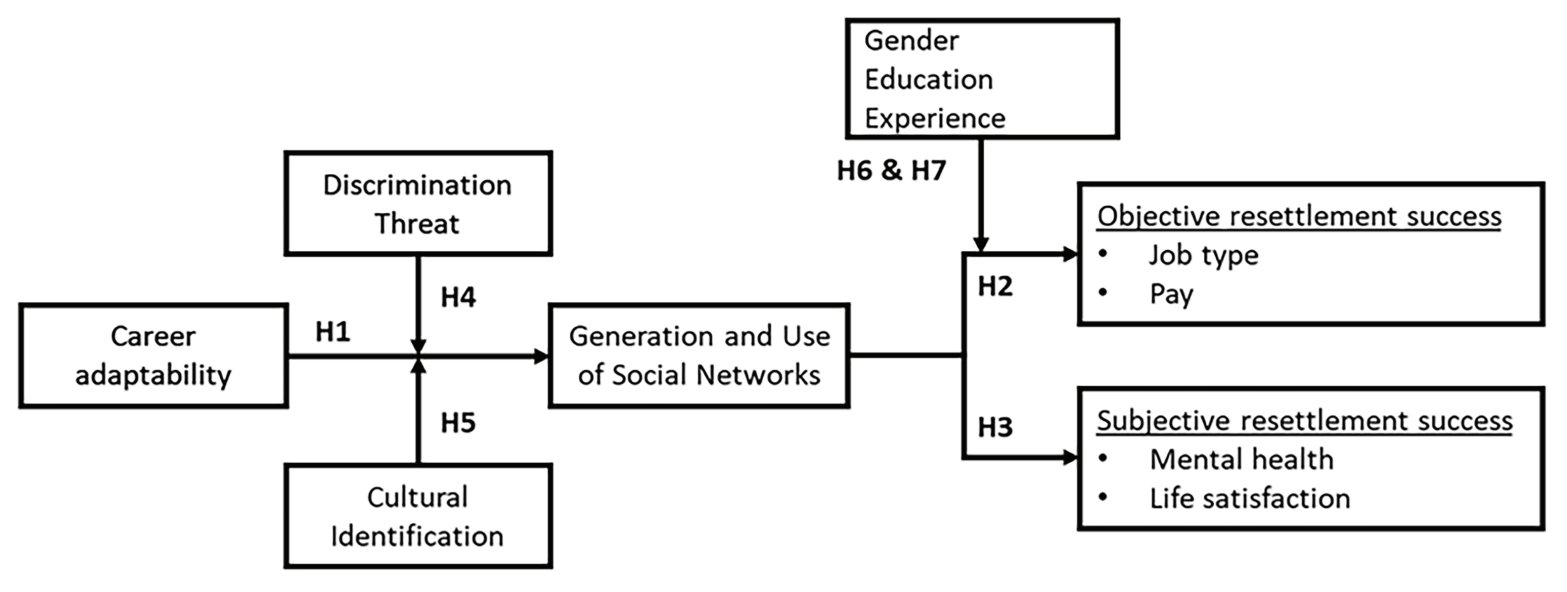
Theoretical model of career adaptability in relation to refugee resettlement success. Adapted from Campion (2018). Cultural identification was excluded from the original model, instead a variable named “host country language ability upon arrival” was included.

The main objective of the study is to test, adapt, and expand a novel model of work resettlement as proposed by Campion (2018). The study focuses on a group of refugees transitioning to a host country that is culturally similar to their home country. This model intends to explain the structural and personal barriers (e.g., discrimination threat) to objective and subjective resettlement. Campion (2018) stated that objective resettlement success is defined as job type and wage, whereas subjective resettlement success is defined according to mental health and life satisfaction. A second important goal of the research is to introduce “cultural identification with the host country” as a moderating variable, which may potentially address the abovementioned uniqueness of Venezuelans who are settling in Colombia. The theoretical model is grounded on two complementary domains, namely, career construction and social networks theory (Figure 1).

Grounded on these domains, the study presents four main contributions. First, addressing the calls for future research on refugees (e.g., Newman et al., 2018), the study empirically tests (combining quantitative and qualitative methodologies) a conceptual model of how refugees search for and secure employment. Although the vast majority of studies on refugees address their integration into a host country labor market from a person-centric approach (Wehrle et al., 2018), the current study intends to integrate contextual factors into the understanding of the career adaptability of refugees and focuses on personal narratives at the same time. Second, the study expands the model of Campion (2018) by testing the role of cultural identification with the host country. Third, the current study differs from the vast majority of studies on refugees by exploring career adaptability in a Venezuelan refugee group relocated in Colombia, where cultural identification is generally assumed to be low. Moreover, the study examines a group of refugees that moved from a home developing country to a host country that is also an emerging economy. Both countries feature a shared history, geography, and language and many other cultural features. Fourth, by investigating the process of refugee resettlement in terms of work life, the study provides a roadmap for key stakeholders, such as refugees themselves, host country agents or organizations, and all possible agents that constitute the refugees’ social networks. Such parties may help these agents to formulate wise initiatives and contribute better to the integration of refugees in the workforce. The current paper presents two studies as follows. Study 1 is a quantitative study where the Campion model of career adaptability is empirically tested, whereas Study 2 is a qualitative study that aims to explore the unexplained statistical relationship between discrimination threat and cultural identification to identify other processes and factors that may be involved beyond the theoretically tested model.

### Career Construction, Career Adaptability, and Generation and Use of Social Networks

The main tenet of career construction theory is that *career* – the development of vocational behavior over time – is a reflection of the course of one’s vocational behavior – a person’s responses toward the selection and adaptation to an occupation – but not the vocation behavior itself (Savickas, 2002). In other words, career construction occurs throughout life and builds upon the actualization of certain developmental tasks, such as reflection on work-related choices, adaptation strategies, and experienced changes. Based on this reflexive exercise, people are transformed into the main *actors* of their *own story* (Savickas, 2002). One important emphasis of career construction theory is career adaptability. It is a psychological construct that informs the self-regulatory behaviors of a person during a job search and, therefore, informs us about individual differences in career trajectories (Campion, 2018). Campion’s model proposes that career adaptability should shape the behaviors and attitudes of refugees in response to employment challenges, such as refugee resettlement, which in this case is translated into finding a job and securing social support. Therefore, career-adaptive refugees are likely to generate and use social networks in their endeavor to find resettlement (e.g., employment opportunities). As such, the study hypothesizes that:

*Hypothesis* 1: Career adaptability is positively related to the generation and use of a social network in the refugee job search process.

### Structural and Personal Barriers for Refugee Resettlement

Campion (2018) model expands the career construction theory by proposing that education, experience, effortful job search, and career adaptability are insufficient to guarantee high-quality employment for job seekers. The argument is that career construction theory predicts that humans create their success; however, this construct also neglects the influence of structural barriers. Therefore, to better understand refugee resettlement success (subjective or objective), considering the intersection between career adaptability and structural barriers is necessary.

As previously mentioned, career adaptive refugees will look for opportunities of social support *via* social network generation and use in their venture into successful resettlement. However, this notion may be a double-edged sword. First, based on the concept of *homophily*, career adaptive refugees are expected to associate with others similar to them (McPherson et al., 2001), compared with native citizens. Second, based on the concept of *ethnic niche employment*, career adaptive refugees are also expected to secure a quick but not high-quality employment. Several interrelated mechanisms explain this exposure to low-quality jobs (e.g., non-recognition of qualifications or race and cultural discrimination), which make refugees concentrate in labor market niches, such as cleaning services, taxi drivers, or care givers (Colic-Peisker and Tilbury, 2006). Third, empirical research shows that ethnic networks in the job search of migrants search have a direct negative effect on job quality of employment because information gathered from such informal sources is low quality (Mahuteau and Junankar, 2008). Thus, although informal sources are important for quickly securing jobs (Yamauchi and Tanabe, 2008), such jobs may be low in quality (Mahuteau and Junankar, 2008). Fourth, career adaptive refugees are expected to secure jobs similar to those of the members of their social network because they prioritize the generation and use of a social network. Indeed, empirical evidence indicates that a social network influences career adaptability (Guan et al., 2015; Garcia et al., 2019). Within the current model of career adaptability framework, this influence is explained by the concept of social expectations, which are primarily translated into strong norms imposed by the social network of members. In turn, this network influences the occupational decisions of refugees (Campion, 2018).

In summary, this study expects that career adaptive refugees will achieve a lower level of objective resettlement success (i.e., wage and job type), for instance, in comparison with the locals. The reason underlying this notion is that the generation and use of a social network with similar people constrains the quality of employment as a result of the low quality of information and social expectations from such a social network. These aspects yield lower pay and lower job types compared with their qualifications. In addition, the model predicts that career adaptive refugees will experience positive psychosocial benefits. This paradox is explained by the generation and use of social networks with similar people, which eventually results in a high likelihood of career adaptive refugees landing a job where similar people are employed. This, in turn, may yield low turnover (Maertz et al., 2003) and high levels of social support, mental health, and job satisfaction. Therefore, this study hypothesizes that:

*Hypothesis* 2: Social network generation and use will mediate the relationship between career adaptability and objective resettlement success.
*Hypothesis* 3: Social network generation and use will mediate the relationship between career adaptability and subjective resettlement success.

### Discrimination Threat

Another important structural barrier for successful refugee resettlement identified by Campion’s model is discrimination threat. Discrimination is defined as “a set of implicit or explicit norms and behaviors that deny some right of a group of people, usually a minority without any power, for possessing a physical, personal, cultural, or social characteristic that has been identified (for the rest of the society) as negative” (Campo-Arias et al., 2015, p. 39). The study on perceived threats by minority groups is a relevant topic at the moment (e.g., Jedinger and Eisentraut, 2020). Thus, the current study focused on discrimination threat, which is also an obstacle of securing a high-quality job type for career adaptive refugees. As a result, this study expects that career adaptive refugees will opt for the generation and use of social networks with similar people and accept jobs similar to them to avoid discrimination threat during the job search process. However, they also receive social support from such social ties. Therefore, this study hypothesizes that:

*Hypothesis* 4: Discrimination threat will moderate the relationship between career adaptability and generation and use of social networks, such that, refugees who perceive higher discrimination threat will more heavily generate and use social networks.

### Cultural Identification

Cultural identification is an indicator of sociocultural adaptation (Sussman, 2000) and refers to the extent to one’s sense of shared values, beliefs, norms, attitudes, meanings, and customs with a group. Campion’s model proposes that language proficiency may help career adaptive refugees to form ties with people beyond similar others. In other words, they are likely to reach out with weak ties. These ties hold non-redundant information about employment and, therefore, refugees may escape from the ethnic niche employment. This tendency results in increased objective resettlement success. However, this study examines refugees with high cultural commonalities with the host country, i.e., speaking the same language (Spanish). This characteristic of the sample in focus leads the current study to employ cultural identification as a proxy variable of one’s connectedness to the new culture instead of language proficiency. Indeed, previous studies indicated that second language acquisition and proficiency (e.g., pronunciation) “is the strongest linguistic marker of a speaker’s cultural identification” (Lybeck, 2002, p. 174). Furthermore, individuals may experience group identification in various forms, and earlier studies illustrated that group identification can alleviate the negative consequences of felt discrimination (Branscombe et al., 1999; Schmid and Muldoon, 2015). As such, the present study hypothesizes the following:

*Hypothesis* 5: Cultural identification will moderate the relationship between career adaptability and the generation and use of social networks, such that career adaptive refugees with high cultural identification with the host country will heavily generate and use social networks.

### Gender

According to Campion’s model, gender represents a personal barrier to refugee resettlement because it constrains and guides certain types of behavior. Campion (2018) provided three main arguments that sustain this general proposition. First, in accordance with the social role theory, people hold gender role beliefs based on sex differences and similarities in behavior, which, in turn, represent social roles for men and women (Eagly and Wood, 2012). Second, career construction theory assumes that career construction occurs when people develop and implement self-concepts. In turn, these concepts are established through the interaction of aptitudes, physical make-up, and satisfactory evaluations from peers or supervisors of performance in work roles (Savickas, 2002). Third, previous research indicates that female and male refugees secure employment that are closely related to their social gender roles, such as babysitters, housekeepers, or construction workers. For instance, Koyama (2015) observed a group of sub-Saharan African women living in New York City and found that the preponderance of these refugees in the food industry may be related with the notion that women belong to the kitchen. This gender role may be shared by people not only at home but also in the host country.

Taken together, social roles constrain and guide behaviors, which, in turn, exert pressure on career adaptive refugees and forces them to accept jobs that may deviate from their self-concept. The result is an alignment with a social role at the expense of the self-concept. Therefore, this study presents the following hypothesis:

*Hypothesis* 6: Gender will moderate the relationship between the generation and use of social networks and objective resettlement success, such that women and men that rely on their network will be more likely to accept lower job status (e.g., cleaning, child care for women, or physically demanding labor for men).

### Education and Experience

As previously mentioned, the non-recognition of qualifications and certifications is a common challenge among refugees. It is a structural barrier for refugee resettlement that is based on a phenomenon called *deskilling* (Campion, 2018). Deskilling occurs when people work in jobs that are lower than their qualifications or when people simply lack the right to work for a certain period of time. This scenario produces a detriment in knowledge, abilities, and skills, which lead to an eventual recertification that is practically unrealistic (Philo et al., 2013; Stewart, 2003). For instance, in the United Kingdom, several legislations have denied working rights to asylum seekers since 1996. However, since 2005, they were allowed to work if they had been waiting for at least 1 year for a decision on their legal status, thus resulting in deskilling (Philo et al., 2013). Campion argued that adaptive refugees with high levels of educated and more career experience, who engage in their social networks to secure employment, are more likely to suffer from deskilling, thus affecting objective resettlement success. Therefore, this study hypothesizes that:

*Hypothesis* 7: Education and experience will moderate the relationship between the generation and use of social networks and objective resettlement success, such that refugees with high levels of education and more work experience, who rely on social networks for job information, will be more likely to experience a greater downward occupational mobility than less-educated refugees who rely on social networks.

## Study 1: Empirical Examination of Campion’s Model

Study 1 aims to test the model of career adaptability in a sample of Venezuelan migrants in Colombia.

## Materials and Methods

### Participants and Procedure

Data (*n* = 81) were collected using a non-probability sampling technique from a group of Venezuelan employees working in Bogotá, Colombia. The average age for all participants was 38.3 years (range = 20–63, *SD* = 11). The two main inclusion criteria were: (1) having Venezuelan citizenship and (2) currently working with an organization in Colombia. Additionally, the study excluded independent or informal workers. A correlational–exploratory design was used and two procedures were considered to identify working Venezuelans in Bogotá. First, the researchers attended a fair organized by a non-governmental organization, whose objective is to improve the quality of life of Venezuelans in the city. During this fair, assistants were approached and given a summary of our research. If they were interested in the study, the researchers then asked for their e-mail address on which to send a link with questionnaires. A total of 124 assistants were approached, out of which 29 were currently unemployed. Out of the remaining 95, 38 were interested in the research and were sent an e-mail. The second procedure involved the snowball sampling technique. The researchers asked Venezuelan students and faculty in our department to inquire with their Venezuelan acquaintances if they were interested in the research. With this technique, a total of 103 e-mails were sent.

Data were collected online using Qualtrics. At the beginning of the survey, employees were informed about the confidentiality of information provided. Within the questionnaire, information about the research project, role as participants, and consent to participate were provided. Before gathering data, the ethics committee from the corresponding department approved the research.

### Variables and Measures

To test Campion’s model of resettlement success we follow her own recommendations about which variables should be included in the model and include a measure intended to assess each of those variables.

#### Career Adaptability

The study employed the career adaptability scale of Savickas and Porfeli (2012) in its Spanish adaptation by Merino-Tejedor et al. (2016). It is a self-report measure with 24 items that ask the participants to rate their career adaptability-related abilities (e.g., “making decisions by myself,” “learning new skills,” and “preparing for the future”). Items were scored on a 5-point Likert scale ranging from 1 (*not strong*) to 5 (*strongest*).

#### Generation and Use of Social Network

The level of generation and use of social networks was measured using the 6-item Lubben Social Network Scale developed by Lubben et al. (2006) and adapted into Spanish by Vilar-Compte et al. (2018). The scale asked respondents to think about the availability of family and friends for support (in terms of numbers). A sample item is “Considering the people to whom you are related either by birth or marriage. How many relatives do you see or hear from at least once a month?” Items were scored according to six response categories ranging from 1 (*none*) to 6 (*nine or more*).

#### Discrimination Threat

This variable was measured *via* the 9-item discrimination threat scale developed by Williams et al. (1997) using the Spanish version adapted by Campo-Arias et al. (2015). The scale measured the frequency of discrimination events. A sample item is “In your daily life, how many times did the following things happen to you…being treated with less courtesy than others?” Items were scored on a 6-point Likert scale ranging from 1 (*never*) to 6 (*almost every day*).

#### Cultural Identification

Cultural identification with the host country was measured using the 4-item scale developed by Schulz and Leszczensky (2016). A translation back-translation process as proposed by Brislin (1980) was followed, and adequate indicators of reliability (composite reliability = 0.95) and validity (AVE = 0.81) were obtained. A sample item is “I feel part of the Colombian culture.” Items were scored on a 7-point Likert scale ranging from 1 (*strongly disagree*) to 7 (*strongly agree*).

#### Gender

Gender was a categorical variable, where 57% of the sample was composed of females.

#### Education and Experience

For *education*, the highest level of education was recorded on a scale of 1–5, in which 1 = primary school, 2 = high school, 3 = technical education, 4 = college, and 5 = postgraduate. In the study, 3.7, 40.7, and 54.3% of the respondents completed education at the high school/diploma level, undergraduate level (university, technical, or technological education), and postgraduate level. For *experience*, the total number of years of work experience was recorded. The mean years of work experience was 14.5 (range = 1–40, *SD* = 11.2).

#### Objective Resettlement Success

Following Campion (2018), job type and pay were assessed. A self-rated occupational status classification (i.e., blue collar, administrative, technical, professional, and managerial) was used for *job type*. The composition of the sample was blue collar = 16%, administrative = 7%, technical = 5%, professional = 57%, and managerial = 15%. In terms of *pay*, a self-report of the current wage in Colombian pesos was employed using five categories ranging from 1 (approximately 330 US dollars, which is the minimum monthly wage in Colombia) to 5 (more than 3,000 US dollars).

#### Subjective Resettlement Success

Following Campion (2018), psychological health and life satisfaction were assessed. The 12-item General Health Questionnaire developed by Goldberg (1978) in the Spanish version by Silla (2007) was used to measure *mental health*. This scale asks the participants to report the frequency in which they have experienced certain events (e.g., “able to concentrate,” “capable of making decisions,” and “lost much sleep”). Items were scored on a 4-point Likert scale ranging from 1 (*less than usual*) to 4 (*much more than usual*). *Life satisfaction* was measured using the 5-item life satisfaction scale developed by Diener et al. (1985) in its Spanish version by Vázquez et al. (2013). A sample item is “I am satisfied with my life.” The items were scored on a 7-point Likert scale ranging from 1 (*strongly disagree*) to 7 (*strongly agree*).

### Statistical Analyses

#### Common Method Biases

All the variables were provided by the same job incumbent and in a single sitting. Thus, to test for common variance effects on the results, Harman’s single-factor test was conducted (see Podsakoff et al., 2003) to test an unconstrained and a zero-constrained model.

#### Structural Equation Model

To test the original model of Campion (2018) and our hypotheses, a structural model was run using AMOS 25 (Arbuckle, 2017). Various fit indices were reported: *χ*^2^/*df* ratio, comparative fit index (CFI), standardized root-mean-square residual (SRMR), and root-mean-square error of approximation (RMSEA). For the *χ*^2^/*df* ratio, a value of 2.0 indicates good fit. For CFI, values higher than 0.90 indicate good fit. For SRMR, values lower than 0.08 indicate good fit. Lastly, a value of 0.05 indicates good fit for RMSEA. The model was considered acceptable if all fit indexes were within the said limits.

## Results

### Descriptive Statistics and Bivariate Correlations

The study reports the means, standard deviations, bivariate correlations, and reliability coefficients for all listed variables in Table 1. Analyses suggest that, in general, all variables have average levels of skewness (1.5) and kurtosis (2.7). However, for career adaptability and discrimination, the values were higher: skewness (−2.30 and 2.47 for adaptability and discrimination, respectively), kurtosis (9.27 and 8.10 for adaptability and discrimination, respectively). The results of the common latent factor test indicated that the unconstrained and zero-constrained models were equal (i.e., *p* > 0.05). Therefore, no specific response bias affecting the assessment model was observed (Gaskin and Lim, 2017).

**Table 1:** Descriptive statistics and bivariate correlations.

	M	SD	1	2	3	4	5	6	7	8	9	10
1. Career adaptability	4.37	0.63	(0.97)									
2. Discrimination threat	1.58	0.72	0.08	(0.91)								
3. Social networks generation and use	3.57	0.98	0.33^**^	−0.32^**^	(0.86)							
4. Psychological health	2.83	0.54	0.38^**^	−0.45^**^	0.33^**^	(0.80)						
5. Life satisfaction	5.04	1.49	0.40^**^	−0.50^**^	0.37^**^	0.58^**^	(0.86)					
6. Cultural identification	5.19	1.50	0.28^*^	−0.39^**^	0.31^**^	0.34^**^	0.55^**^	(0.95)				
7. Gender	–	–	0.04	−0.11	0.13	0.29^**^	0.15	0.05	–			
8. Education	4.36	0.87	0.22	−0.38^**^	0.18	0.17	0.30^**^	0.29^**^	−0.02	–		
9. Pay	2.65	1.30	0.05	−0.35^**^	0.16	0.19	0.35^**^	0.14	0.25^*^	0.49^**^	–	
10. Job type	–	–	0.16	−0.44^**^	0.25^**^	0.25^*^	0.30^**^	0.13	0.24^*^	0.56^**^	0.57^**^	
11. Experience	14.46	11.18	0.20	−0.20	0.31^**^	0.16	0.30^**^	0.21	0.11	0.37^**^	0.27^*^	0.37^**^

*n* = 81. Reliability coefficients are enclosed in parentheses. The frequency of gender and percentages of different job types are reported in the Method section of Study 1.

* *p* < 0.05;

** *p* < 0.01 (two tailed).

### Test of Hypotheses

**Figure 2:**
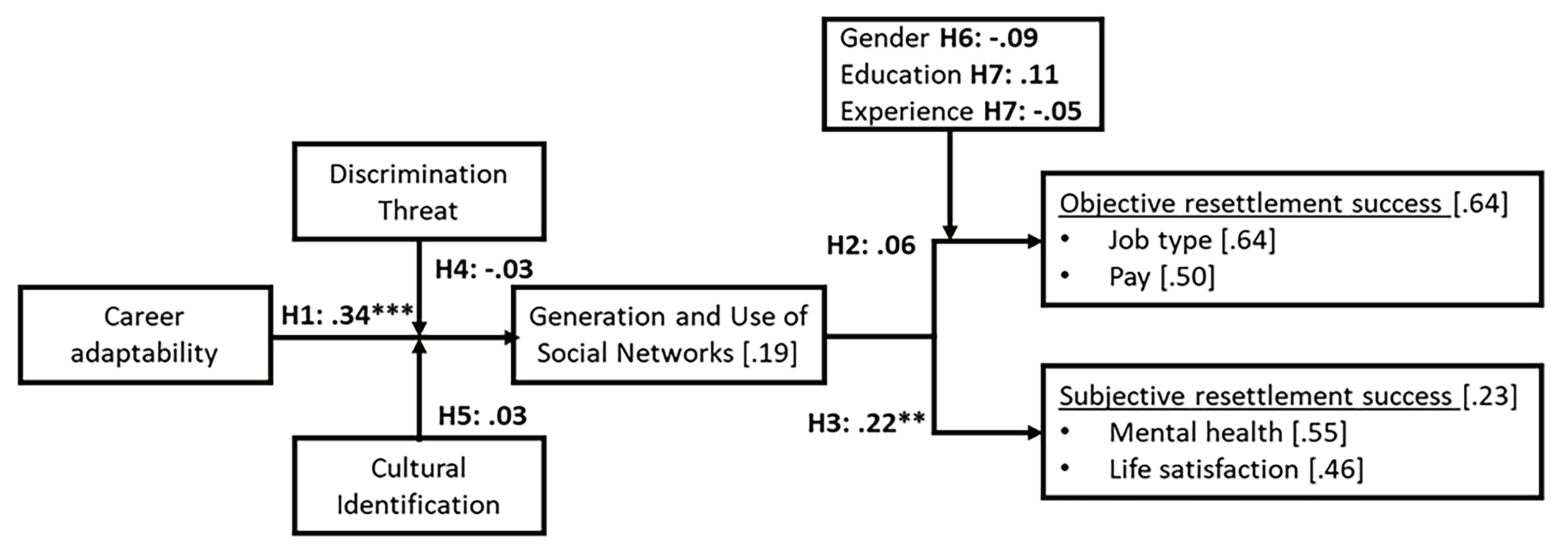
Theoretical model of career adaptability in relation to refugee resettlement success. Adapted from Campion (2018). ^**^p < 0.01 and ^***^p < 0.001. *R*^2^ values are enclosed in brackets for each outcome variable.

To test the model of Campion (2018) and our hypotheses, a structural equation model was run. Results of the global test indicate adequate fit (*χ*^2^/*df* ratio = 1.643, CFI = 0.92, SRMR = 0.06, RMSEA = 0.09). Figure 2 presents a graphical depiction of the model, which includes the significant standardized loadings for each path of the hypotheses. As seen in Figure 2, only two hypotheses were sustained, namely, Hypothesis 1 (career adaptability was positively related to the generation and use of social networks) and Hypothesis 3 (generation and use of social networks mediate the relationship between career adaptability and subjective resettlement success; indirect effect = 0.22, *p* < 0.01). No evidence was found for the mediating role of the generation and use of social networks in the relationship between career adaptability and objective resettlement success (Hypothesis 2), the moderating role of discrimination threat (Hypothesis 4), or cultural identification (Hypothesis 5) on the relation between career adaptability and generation and use of social networks. Furthermore, the moderating role of gender (Hypothesis 6) or education and experience (Hypothesis 7) on the relationship between generation and use of social networks and objective resettlement success was unsupported. Given these results, certain modifications were made in the original model of Campion (2018). Moreover, *post hoc* analyses were performed to better understand the role of key variables.

### *Post hoc* Analyses

**Figure 3:**
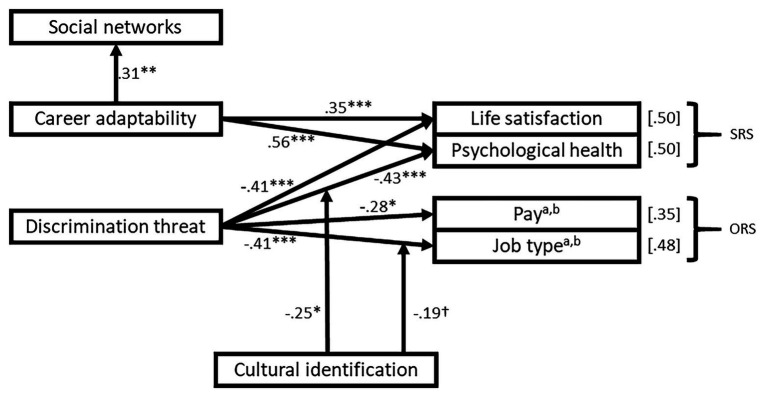
Model of resettlement success for Venezuelans in Colombia. SRS, subjective resettlement success; ORS, objective resettlement success. Control variables: ^a^Gender (0.19^*^ for job type, 0.21^*^ for pay), ^b^Education (0.43^***^ for job type, 0.44^***^ for pay). ^†^*p* < 0.10, ^*^p < 0.05, ^**^p < 0.01, and ^***^p < 0.001. *R*^2^ values are enclosed in brackets for each outcome variable.

As previously mentioned, the results of Study 1 suggested that Campion’s model should be updated given that several hypotheses were unsupported. Therefore, *post hoc* analyses were conducted to test the most critical factors for refugee resettlement success, for instance, focusing on relevant moderators, such as cultural identification. The term refers to an extension of Campion’s model of refugee resettlement and potentially, an important contribution of this paper. *Post hoc* analyses suggested four main updates. First, career adaptability is related to the generation and use of social networks. Second, gender, education, and experience were excluded as moderators and designated as control variables. Third, career adaptability and discrimination threat may have a direct relationship on the indicators of objective and subjective resettlement success. Fourth, cultural identification may be a moderator of the relationship between discrimination threat and cultural identification and the indicators of objective and subjective resettlement success. This model showed adequate overall fit, with the lowest *χ*^2^/*df* ratio, SRMR, RMSEA, and highest CFI values (Table 2). Figure 3 presents a graphical depiction of the modified model, which includes the significant standardized loadings.

**Table 2:** Results of confirmatory factor analysis.

Model	*χ*^2^	*Df*	*χ*^2^/*df* ratio	SRMR	RMSEA	CFI
Original model	76	46	1.643	0.06	0.09	0.92
Adapted model	21	16	1.28	0.07	0.06	0.98

SRMR, standardized root-mean-square; RMSEA, root-mean-square error of approximation; CFI, comparative fit index.

## Discussion

Study 1 is quantitative in nature and aims to empirically test the Campion (2018) model of career adaptability and our hypotheses. The results indicated that although the global model of resettlement success showed adequate levels of fit, five of the proposed seven specific paths demonstrated non-significant results. For the significant paths, the study found that social networks play a mediating role in the relationship between career adaptability and subjective but not objective indicators. In accordance with the concepts of homophily and ethnic niche employment, the study further found that, although social networks are related to career adaptability, they do not play a key role in the objective resettlement success of migrants. However, social networks provide shelter and emotional support, which, in turn, is translated into improved levels of psychological health and life satisfaction. In conclusion, a key finding from Study 1 is that networks, in contrast to non-refugee samples, do not matter for settlement objective outcomes. This finding is notable and interesting because the bulk of the literature suggests that social capital is advantageous to career success.

Furthermore, the results indicated that resettlement success (objective and subjective) is highly influenced by discrimination threat. However, the results also revealed that cultural identification can buffer this negative effect. Therefore, one key conclusion from Study 1 suggests that cultural identification can help lessen the negative effects of discrimination threat on objective and subjective resettlement success of Venezuelan refugees in Colombia. Therefore, cultural identification may be a boundary condition and a core contribution of the paper. In conclusion, *post hoc* analyses suggest the need to update and extend Campion’s model of career adaptability, where cultural identification may be an important boundary condition.

At the same time, Study 1 led to further questions about the resettlement process of Venezuelan refugees in Colombia given that social networks did not influence resettlement success. Study 2, which uses a qualitative method and in-depth interviews with Venezuelan refugees, was developed to better understand the role of several of the relationships identified in Study 1 and because the sample size could not be expanded to sufficiently explore Campion’s model.

## Study 2: Qualitative Iteration of Campion’s Model

The study tested the model of career adaptability in relation to refugee resettlement success (Campion, 2018), which, to the best of our knowledge, was suggested but not empirically tested. The model was tested in a sample of Venezuelan refugees who have settled in Colombia. Thus far, three main findings are notable. First, career adaptability and the generation and use of social networks play important roles in subjective but not objective resettlement success. Second, neither discrimination threat nor cultural identification plays an important role in resettlement success (objective and subjective). Third, gender, education, and experience may not be boundary conditions in terms of resettlement success. Drawing on these findings and considering the multi-facet nature of the topic (Syed, 2008; Zikic et al., 2010; Zikic, 2015; Zikic and Richardson, 2016), the research question was re-examined using a qualitative method. This scheme could enable the study to further shed light on the context, thus responding to calls for a more contextualized perspective in career studies (Chudzikowski and Mayrhofer, 2011; Al Ariss et al., 2012) and contribute to a broader literature. In the following section, the findings from the qualitative Study 2 are described, which focused on the three quantitative findings.

## Materials and Methods

Mixed methods, specifically the case of using qualitative research methods after quantitative research, is recommended for a follow-up and to explore unexplained statistical results. In the case of the present study, such results pertain to the surprising outcome of cultural identification as an independent variable and an antecedent to the successful resettlement of Venezuelan refugees in Colombia. As demonstrated, the deductive model that was tested (Campion’s model) required in-depth understanding of cultural identification as a variable. Therefore, the study selected the inductive method to complement the first one on the premise that “by building theory inductively, research based on qualitative data offers insights that challenge taken for granted theories and expose new theoretical directions” (Bansal et al., 2018, p. 1189).

### Participants

Eight participants (four women and four men) from Study 1 volunteered for face-to-face interviews held at the university. Interviews were semi-structured and used the main findings of Study 1 as a guide.

### Procedure

Building on the quantitative findings, the qualitative study aimed to examine the support and threat mechanisms that played an important role in career adaptation processes according to the unique case of each participant. The study was mainly interested in a better understanding of the stages of the adjustment process because Campion’s model refers to a “process” of resettlement success. The current study especially focused on the driving and restricting forces confronted during this process and their effects on the identity and career adjustment experiences of individuals. Focusing on the processes enables the identification of the emergence, changes, and unfolding nature of the phenomena over time without overlooking “the temporal structure of social practices and the uncertainty and urgencies that are inherently involved in them” (Langley et al., 2013, p. 4). This approach allowed us to investigate the micro, meso, and macro factors that interact over time, such as education, gender, discrimination, support from social networks, legal constraints, and others emphasized by participants. In this manner, a useful heuristic evaluation can be provided for extending the research on career adaptation processes.

With this goal in mind, the interviewees were instructed to draw a journey map to trace significant events over time. They were given 10–15 min to draw their personal journey maps. Once ready, the interview was conducted based on their personal drawings. The interviewers had a predetermined set of discussion topics for the interview. The order in which these topics were addressed is dependent on the drawings of the participant for each session. Drawing a journey map enabled the interviewees to better remember facts, decisions, people, and emotions experienced during the transition process. The time given for drawing encouraged them to reflect on their resettlement processes. This method ensured that their answers were deep and rich in detail. The drawings “generate(d) deep insight by going beyond rational cognitive ways of knowing and providing new ways of understanding people’s real lived experiences and views” (van der Vaart et al., 2018, p. 1). In this manner, values, beliefs, and feelings, were uncovered, which played an important role in the understanding of social realities (Schwartz-Shea and Yanow, 2012).

## Results

The interviewees cited that having qualifications and related work experience were key factors to getting a job.

“What has worked for me is that I’ve had the technical knowledge they were looking for.” (I5_110419)^1^

“…these were highly qualified jobs and I had the corresponding background.” (I6_120419)

“I have two degrees, but I don’t have one of the (physical) diplomas with me, so I can only apply to the jobs that are related to the diploma I’ve got here with me.” (I3_040419).

In addition, the interviewees mentioned that their curriculum vitae opened doors when applying for new jobs. All interviewees reported finding a job by applying *via* job search websites. However, at the same time, all interviewees mentioned having landed a job because of another person who referred them.

When asked regarding the importance of social networks in finding a job, the majority of interviewees felt disadvantaged compared with Colombians because the locals of the host country had larger networks and a better understanding of how to use such networks.

“Here, you’re no one (*un Don Nadie*).” (I1_040419)

Even if the interviewees knew people from their Venezuelan social networks, they felt that they were unable to help.

“I do know many Venezuelans who have a job here in Colombia, but they’re struggling in the same way I am; you can’t ask them for support.” (I1_040419)

Most interviewees opted to migrate to Colombia because they have Colombian relatives.

“I came to Colombia because my parents are Colombians. I had the chance to get the (Colombian) nationality and in that way, I wouldn’t depend on a visa to find a job nor to stay.” (I6_120419)

“My sister lives in the US, but I preferred Colombia. My boyfriend at that time was Colombian.” (I1_040419)

“I would’ve liked living in Medellin, but my husband has some relatives who live in Bogota.” (I5_110419)

The interviewees mentioned that these relatives are a good first support upon arrival. Afterward, however, finding a place to live and a better-fitting job is dependent on the interviewees.

Although family is the main emotional support mechanism and frequently provides entry to a new network (at least to a critical person even if not to a large network). For those who left their relatives in Venezuela, this factor is a driving and a restraining force. It is a driving force because they are working with the goal of providing help to those they have left behind.

“At the moment I economically support four households who are family still living in Venezuela.” (I6_120419)

“I have to keep moving, because I haven’t reached the point at which I can economically help my family in Venezuela.” (I5_110419)

However, the interviewees keep thinking of them and feel a mixture of emotions and may be even feeling slightly guilty, which is exhaustive and thus restrains adjustment and success.

“70% of my mind is over there (in Venezuela).” (I3_040419)

“Being (a) workaholic is good because it’s better to keep my mind busy at work rather than thinking of Venezuela.” (I5_110419)

In relation to cultural identification, the interviewees mentioned that although Colombians and Venezuelans seem very similar, the opposite is true in reality.

“Deep inside, we’re really different. It’s difficult to make friends. You don’t know when you can trust someone as a friend.” (I5_110419).

Moreover, the interviewees mentioned that acting according to Colombian manners is important because Colombians may feel uncomfortable with Venezuelan manners.

“In Venezuela we make more jokes, we like *chaquetear*. I’ve worked in other Latin American countries and I’ve learned to adjust my manners. This has helped me when applying to jobs in Colombia. When I come across other Venezuelans at work, I notice their behavior bothers some people.” (I6_120419)

“Here I behave like a Colombian.” (I1_040419)

Finally, the interviewees mentioned that people who found non-qualified jobs, such as waiter/waitress or a delivery person, feel more discriminated against.

“When you’re in need, you end up accepting any position, even if you’re much more qualified. Some companies take advantage of your situation, so you feel discriminated.” (I3_040419)

In contrast, the higher the rank in an organization, the easier it is to move toward better positions.

“Once they start knowing you, they recommend your job.” (I6_120419)

## Discussion

Study 2 is a qualitative analysis that enabled us to explore structural and personal barriers between career adaptability and resettlement success in detail, which Campion (2018) identifies as the key contribution of the model. The qualitative study allowed us to go beyond a certain set of barriers and collect information about different types of barriers. This process enabled the revelation of which factors the interviewees emphasized, that is, cultural identification. A few of the major consistencies in the responses of the interviewees were (a) having higher qualifications and work experience, (b) limited utility of social networks with similar others (i.e., other Venezuelans), (c) low cultural identification despite sharing cultural commonalities (e.g., language), and (d) downward employment. As demonstrated, several of the propositions in Campion’s model are reflected in the general results of the qualitative examination. However, similar to the main conclusion of Study 2, a proposition from Campion’s model, which is not supported by qualitative Study 2, suggests that social networks are important for new refugees but do not play a key role in resettlement success. At this point, the study discusses vis-à-vis the quantitative and qualitative results.

## General Discussion

Grounded on two complementary theoretical frameworks, namely, career construction and social networks theory, the main objective of the study was to provide empirical evidence of the model of career adaptability in refugee resettlement success as proposed by Campion (2018). To achieve this objective, the model and our hypotheses were tested in a sample of Venezuelan refugees in Colombia. This aspect renders the current study distinct from the vast majority of existing research because of the focus on a group of refugees transitioning to a country that shares common cultural heritage with their home country. Moreover, the study aimed to expand the model by introducing “cultural identification with the host country” as a relevant boundary condition. Given the disappointing results from quantitative Study 1, qualitative Study 2 was conducted to further explore the processes of career adaptability and refugee resettlement success.

### Key Results

The main result from Studies 1 and 2 is that, in contrast to the majority of literature suggesting that social capital is advantageous to career success, networks do not play a key role in refugee objective resettlement success. Study 2 further shows that the impact of a social network is relatively spontaneous and unpredictable. Personal narratives revealed that the major actors in the resettlement of refugees were usually themselves. At critical moments, such as in making the decision to leave Venezuela and in the process that follows, the interviewees stand out as the main agents. The influence of social ties in this process is frequently through coincidence and by a self-initiated, voluntary help-giver. The expansion of social ties is deemed random and mostly by the intervention of a volunteering person (i.e., family members and total strangers) but not necessarily with the initiation of the main actor (refugee). This finding opens another direction for examining refugee resettlement not only through the lens of structural and psychological dimensions but also the process dimension.

Campion (2018) suggested that the creation of a network contributes to better mental health as well as increases life satisfaction. Therefore, the current study suggests a deliberate reconsideration of this because the findings regarding the positive effects of social networks are relatively equivocal. In Study 1, evidence supporting Campion was found; however, results from Study 2 are less clear. The interviewees mentioned that having a job brings them peace and stability. As one interviewee stated: “I feel more adapted now. I’ve got a job and I see my career has some future.” However, the majority of the interviewees emphasized that they are experiencing stress and sadness due to their dual social network memberships. Although they are building a career and life in a new country within a new social network, their ties in Venezuela continue to coexist. The interviewees pointed out the emotional downsides (i.e., feeling sad, guilty, and worried) of strong connections in the country of origin, thus creating a psychological dichotomy. Lastly, the findings suggest the need for further exploration of the refugees’ understanding of their networks and the mechanism they use to maneuver through multiple networks.

Moreover, the results from Studies 1 and 2 confirm that discrimination threat is a major barrier that Venezuelan refugees face in Colombia because it hinders objective and subjective resettlement success. However, the results also demonstrated that cultural identification may help them cope with certain negative effects of discrimination because such effects empower the individual through a better understanding and interpretation of the values and practices of the host work environment. Therefore, one important contribution of the study is updating and extending Campion’s model of refugee resettlement. The study highlighted cultural identification as an important boundary condition of successful refugee resettlement. Among the structural and personal barriers addressed in Campion (2018) model, language fluency was excluded from the quantitative design because it did not apply to the current sample, which included Colombians and Venezuelans in the same cultural cluster (Hofstede, 2001; House et al., 2004). However, the participants in Study 2 emphasized subtle differences in Venezuelan and Colombian Spanish. One of the most successful participants career-wise specifically mentioned his careful use of language (i.e., jokes and specific uses of words). These subtle nuances may play an important role in the refugees’ fit and adaptation processes, at least with regard to their self-perception about how well they fit, even within the “same language” context. Identifying that subtle cultural nuances (e.g., use of specific words, jokes, and expressions) play an important role toward a successful career adaptability renders the results interesting and is the main lesson learned from the study. The notion applies even for refugees moving to a host country that shares cultural commonalities with their home country. In this manner, the study sheds light on the research on career adaptability by demonstrating that cultural identification is not only relevant in refugees transitioning to a host country that does not share many cultural commonalities with the home country (e.g., Eggenhofer-Rehart et al., 2018) but also with countries that are culturally similar.

Finally, although Campion’s model does not focus on soft-skills or traits, the interviews revealed skills considered critical throughout the job search and career adaptation processes. The interviewees are open to new experiences and bravely take the initiative for an unknown future. Many of them started businesses or changed the field in which they work, but the commonality is that they willingly embraced change. Therefore, the study recommends that future research should investigate soft skills, such as personal initiative, courage, resilience, and entrepreneurship intentions.

### Limitations

Four major drawbacks limited the present research. First, in Study 1, the self-reported measures could represent a source of bias as social desirability. Second, we asked each participant to share his/her perceptions, attitudes, behaviors, and demographics. Furthermore, the study used questionnaires to collect all data. These aspects may lead to problems of common method variance. To show discriminant validity, Herman’s single-factor test was performed, which suggested that common method variance was not a problem regarding the data. Third, the small sample size prevented the generalization of the results to the population of Venezuelans working in Colombia or from running additional analyses (e.g., multi-group). Fourth, Study 2 was not an independent qualitative study, but one that was designed to test the findings and limitations of the quantitative examination. Thus, Study 2 was exploratory and descriptive but not fully explanatory. Considering the limitations, the researchers recommend caution in the overall interpretation of the results.

### Implications for Research and Practice

Despite the limitations, the study proposes that the gains related to the understanding of the resettlement process in refugees outweigh the limitations. For research, although the home and host countries share a common language and are in the same cultural cluster, as in the GLOBE project (House, et al., 2004), deepening our knowledge about the cultural identification process is important given that the literature on resettlement (e.g., Eggenhofer-Rehart et al., 2018; Pajic et al., 2018) typically focuses on comparisons between highly distant cultures (i.e., Arabic and European) and heavily overlooks similar cultures. Particularly, the findings from Study 2 indicate that key customs and “micro-cultural differences” may provide an important avenue for future studies. Thus, the study recommends that focus should be given to refugee resettlement, cultural identification as a boundary condition should be examined, and the different layers and aspects of culture should be taken into account to gain a better understanding of the career adaptation process of refugees. Moreover, a clear theoretical implication is that, apart from considering the relevance of career adaptability, cultural identification should be considered as an important boundary condition for an in-depth understanding of career construction and individual differences in career trajectories.

Campion (2018) argued that the career adaptability of refugees does not prioritize the objective indicators of resettlement success because they frequently experience downward occupational mobility. The responses of the interviewees and the quantitative results are in accordance with this notion. Most of the interviewees have higher qualifications than required by their job. Yet, they do not hold a position at the same level as the one they held in Venezuela before leaving. Nearly all interviewees expressed a pre-acceptance of being a misfit (i.e., accept a low-level position) with the hope of advancement in time. Grounding in this replication of Campion’s model, the current study further suggests the examination of expectations versus reality of refugees for a better understanding of their psychological processes.

In addition, the career adaptability of refugees, personality, and psychological capital could provide a fruitful basis for future research for investigating the major strengths of refugees in their resettlement success. In addition, a longitudinal examination of how perceptions about being a misfit evolve over time will be important for further investigation.

Finally, an important lesson is presented for practitioners: the role of cultural identification in the subjective resettlement process, as refugees will better know the customs of the host country (i.e., what is expected and what is considered taboo), facilitates the processes of adaptation, thus influencing life, job satisfaction, and psychological health. In the study, this aspect indicates that Venezuelans will gain better acceptance from the host country locals and suffer less discrimination threat. Furthermore, it can be useful for training programs for newcomers, where the customs of the host country can be taught in advance to enable refugees to better handle the adaptation process.

## Data Availability Statement

The raw data supporting the conclusions of this article will be made available by the authors, without undue reservation.

## Ethics Statement

The studies involving human participants were reviewed and approved by El Comite de Investigacion y Etica de la Facultad de Ciencias Economicas y Administrativas/Facultad de Ciencias Economicas y Administrativas ‐ Decanatura. The patients/participants provided their written informed consent to participate in this study.

## Author Contributions

YA: conceptualization, writing the original draft and review, editing and designing the methodology, and project administration. JB: conceptualization, writing the review, editing and designing the methodology, data collection, and formal analyses. AK: conceptualization, writing the review, editing, and data collection. JP-O: conceptualization, writing the review and editing. MR-M: conceptualization, writing the review, editing and designing the methodology. All authors contributed to the article and approved the submitted version.

### Conflict of Interest

The authors declare that the research was conducted in the absence of any commercial or financial relationships that could be construed as a potential conflict of interest.

## Footnotes

^1^We used the code Ix_date to cite the statement of the interviewees. Ix refers to interviewee number x. The date corresponds to the date on which the interview was conducted.

## References

[ref1] Al Ariss A. Koall I. Özbilgin M. Suutari V. (2012). Careers of skilled migrants: towards a theoretical and methodological expansion. J. Manag. Dev. 31, 92–101. 10.1108/02621711211199511

[ref2] Arbuckle J. L. (2017). “IBM® SPSS® AmosTM 25 User’s Guide,” Amos 25 User’s Guide, IBM Corp.

[ref3] Bansal P. Smith W. K. Vaara E. (2018). New ways of seeing through qualitative research. Acad. Manag. J. 61, 1189–1195. 10.5465/amj.2018.4004

[ref4] Baranik L. E. Hurst C. S. Eby L. T. (2018). The stigma of being a refugee: a mixed-method study of refugees’ experiences of vocational stress. J. Vocat. Behav. 105, 116–130. 10.1016/j.jvb.2017.09.006

[ref5] Branscombe N. R. Schmitt M. T. Harvey R. D. (1999). Perceiving pervasive discrimination among African Americans: implications for group identification and well-being. J. Pers. Soc. Psychol. 77, 135–149. 10.1037/0022-3514.77.1.135

[ref6] Brislin R. W. (1980). “Translation and content analysis of oral and written material” in Handbook of cross-cultural psychology. Vol. 2 eds. Triandis H. C. Berry J. W. (Boston: Allyn and Bacon), 389–444.

[ref7] Campion E. D. (2018). The career adaptive refugee: exploring the structural and personal barriers to refugee resettlement. J. Vocat. Behav. 105, 6–16. 10.1016/j.jvb.2017.10.008

[ref8] Campo-Arias A. Herazo E. Oviedo H. C. (2015). Escala de discriminación en la vida contidiana: Consistencia y estructura interna en estudiantes de medicina. Revista Médica de Risaralda 21, 39–42.

[ref53] Chen X. Liu X. Yu W. Tan A. Fu C. Mao Z. (2019). Association between cross-cultural social adaptation and overseas life satisfaction among chinese medical aid team members (CMATMs) in Africa. Int. J. Environ. Res. Public Health 16:1572. 10.3390/ijerph16091572 PMC6539535 31064049

[ref9] Chudzikowski K. Mayrhofer W. (2011). In search of the blue flower? Grand social theories and career research: the case of Bourdieu’s theory of practice. Hum. Relat. 64, 19–36. 10.1177/0018726710384291

[ref10] Colic-Peisker V. Tilbury F. (2006). Employment niches for recent refugees: segmented labour market in twenty-first century Australia. J. Refug. Stud. 19, 203–229. 10.1093/jrs/fej016

[ref12] Diener E. Emmons R. A. Larsen R. J. Griffin S. (1985). The satisfaction with life scale. J. Pers. Assess. 49, 71–75. 10.1207/s15327752jpa4901_13 16367493

[ref13] Eagly A. H. Wood W. (2012). “Social role theory” in Handbook of theories of social psychology. eds. Van Lange P. A. M. Kruglanski A. W. Tory Higgins E. (United Kingdom: SAGE Publications Ltd), 458–476.

[ref14] Eggenhofer-Rehart P. M. Latzke M. Pernkopf K. Zellhofer D. Mayrhofer W. Steyrer J. (2018). Refugees’ career capital welcome? Afghan and Syrian refugee job seekers in Austria. J. Vocat. Behav. 105, 31–45. 10.1016/j.jvb.2018.01.004

[ref70] Garcia P. R. J. M. Restubog S. L. D. Ocampo A. C. Wang L. Tang R. L. (2019). Role modeling as a socialization mechanism in the transmission of career adaptability across generations. J. Vocat. Behav. 111, 39–48. 10.1016/j.jvb.2018.12.002

[ref15] Gaskin J. Lim J. (2017). CFA Tool. Amos Plugin.

[ref16] Gericke D. Burmeister A. Löwe J. Deller J. Pundt L. (2018). How do refugees use their social capital for successful labor market integration? An exploratory analysis in Germany. J. Vocat. Behav. 105, 46–61. 10.1016/j.jvb.2017.12.002

[ref54] Giorgi G. Lecca L. I. Ariza-Montes A. Di Massimo C. Campagna M. Finstad G. L.. (2020). The dark and the light side of the expatriate’s cross-cultural adjustment: a novel framework including perceived organizational support, work related stress and innovation. Sustainability 12:2969. 10.3390/su12072969, PMID: 16921004

[ref17] Goldberg D. (1978). Manual of the general health questionnaire. Windsor: NFER Publishing.

[ref71] Guan Y. Wang F. Liu H. Ji Y. Jia X. Fang Z.. (2015). Career-specific parental behaviors, career exploration and career adaptability: a three-wave investigation among chinese undergraduates. J. Vocat. Behav. 86, 95–103. 10.1016/j.jvb.2014.10.007, PMID: 16921004

[ref18] Hofstede G. (2001). Culture’s consequences: Comparing values, behaviors, institutions and organizations across nations. Thousand Oaks, CA: SAGE Publications, Inc.

[ref19] House R. J. Hanges P. J. Javidan M. Dorfman P. W. Gupta V. (2004). Culture, leadership and organizations: The GLOBE study of 62 societies. Thousand Oaks, CA: SAGE Publications.

[ref20] Jedinger A. Eisentraut M. (2020). Exploring the differential effects of perceived threat on attitudes toward ethnic minority groups in Germany. Front. Psychol. 10:2895. 10.3389/fpsyg.2019.02895, PMID: 31969850 PMC6960202

[ref21] Knappert L. Kornau A. Figengül M. (2018). Refugees’ exclusion at work and the intersection with gender: insights from the Turkish-Syrian border. J. Vocat. Behav. 105, 62–82. 10.1016/j.jvb.2017.11.002

[ref22] Koyama J. (2015). Constructing gender: refugee women working in the United States. J. Refug. Stud. 28, 258–275. 10.1093/jrs/feu026

[ref23] Langley A. Smallman C. Tsoukas H. Van de Ven A. H. (2013). Process studies of change in organization and management: unveiling temporality, activity, and flow. Acad. Manag. J. 56, 1–13. 10.5465/amj.2013.4001

[ref24] Lubben J. Blozik E. Gillmann G. Iliffe S. von Renteln Kruse W. Beck J. C.. (2006). Performance of an abbreviated version of the Lubben social network scale among three European community-dwelling older adult populations. Gerontologist 46, 503–513. 10.1093/geront/46.4.503, PMID: 16921004

[ref25] Lybeck K. (2002). Cultural identification and second language pronunciation of American in Norway. Mod. Lang. J. 86, 174–191. 10.1111/1540-4781.00143

[ref26] Maertz C. P. Stevens M. J. Campion M. A. (2003). A turnover model for the Mexican maquiladoras. J. Vocat. Behav. 63, 111–135. 10.1016/S0001-8791(02)00023-4

[ref27] Mahuteau S. Junankar P. N. (2008). Do migrants get good jobs in Australia? The role of ethnic networks in job search. Econ. Rec. 84, S115–S130. 10.1111/j.1475-4932.2008.00488.x

[ref28] McPherson M. Smith-Lovin L. Cook J. M. (2001). Birds of a feather: homophily in social networks. Annu. Rev. Sociol. 27, 415–444. 10.1146/annurev.soc.27.1.415

[ref29] Merino-Tejedor E. Hontangas P. M. Boada-Grau J. (2016). Career adaptability and its relation to self-regulation, career construction, and academic engagement among Spanish university students. J. Vocat. Behav. 93, 92–102. 10.1016/j.jvb.2016.01.005

[ref11] Migración Colombia (2019). Más de un millón 260 mil venezolanos se encuentran radicados en el país. Available at: https://www.migracioncolombia.gov.co/noticias/mas-de-1-millon-260-mil-venezolanos-se-encuentran-radicados-en-el-pais-director-de-migracion-colombia (Accessed May 5, 2019).

[ref30] Newman A. Bimrose J. Nielsen I. Zacher H. (2018). Vocational behavior of refugees: how do refugees seek employment, overcome work-related challenges, and navigate their careers? J. Vocat. Behav. 105, 1–5. 10.1016/j.jvb.2018.01.007

[ref31] Pajic S. Ulceluse M. Kismihók G. Mol S. T. den Hartog D. N. (2018). Antecedents of job search self-efficacy of Syrian refugees in Greece and the Netherlands. J. Vocat. Behav. 105, 159–172. 10.1016/j.jvb.2017.11.001, PMID: 29615827 PMC5873528

[ref32] Philo G. Briant E. Donald P. (2013). Bad news for refugees. London: Pluto Press.

[ref33] Podsakoff P. M. MacKenzie S. B. Lee J. -Y. Podsakoff N. P. (2003). Common method biases in behavioral research: a critical review of the literature and recommended remedies. J. Appl. Psychol. 88, 879–903. 10.1037/0021-9010.88.5.879, PMID: 14516251

[ref34] Savickas M. L. (2002). “Career construction: a developmental theory of vocational behavior” in Career choice and development. ed. D. Brown and Associates (San Francisco, CA: Jossey-Bass), 149–205.

[ref35] Savickas M. L. Porfeli E. J. (2012). Career adapt-abilities scale: construction, reliability, and measurement equivalence across 13 countries. J. Vocat. Behav. 80, 661–673. 10.1016/j.jvb.2012.01.011

[ref36] Schmid K. Muldoon O. T. (2015). Perceived threat, social identification, and psychological well-being: the effects of political conflict exposure. Polit. Psychol. 36, 75–92. 10.1111/pops.12073

[ref37] Schulz B. Leszczensky L. (2016). Native friends and host country identification among adolescent immigrants in Germany: the role of ethnic boundaries. Int. Migr. Rev. 50, 163–196. 10.1111/imre.12163

[ref38] Schwartz-Shea P. Yanow D. (2012). Interpretive research design. New York: Routledge.

[ref56] Silla I. (2007). Trabajo temporal, inseguridad laboral percibida y sus implicaciones. Factores piscosociales intervinientes [Unpublished doctoral dissertation]. Universitat de Valenc.

[ref55] Stewart E. (2003). A bitter pill to swallow: obstacles facing refugee and overseas doctors in the UK. Geneva, Switzerland Available at: https://www.unhcr.org/publ/RESEARCH/3fbb94a32.pdf

[ref39] Sussman N. M. (2000). The dynamic nature of cultural identity throughout cultural transitions: why home is not so sweet. Personal. Soc. Psychol. Rev. 4, 355–373. 10.1207/S15327957PSPR0404_5

[ref40] Syed J. (2008). Employment prospects for skilled migrants: a relational perspective. Hum. Resour. Manag. Rev. 18, 28–45. 10.1016/j.hrmr.2007.12.001

[ref42] United Nations High Commissioner for Refugee (2017). Global Report 2017. Regional Summaries. The Americas. Available at: https://www.unhcr.org/publications/fundraising/5b30b9857/unhcr-global-report-2017-americas-regional-summary.html (Accessed November 5, 2019).

[ref43] United Nations High Commissioner for Refugee (2019). Figures at a Glance. Available at: https://www.unhcr.org/figures-at-a-glance.html (Accessed November 5, 2019).

[ref44] van der Vaart G. van Hoven B. Huigen P. P. P. (2018). Creative and arts-based research methods in academic research. Lessons from a participatory research project in the Netherlands. Forum Qual. Sozialforschung 19. 10.17169/fqs-19.2.2961

[ref45] Vázquez C. Duque A. Hervás G. (2013). Satisfaction with life scale in a representative sample of Spanish adults: validation and normative data. Span. J. Psychol. 16:E82. 10.1017/sjp.2013.82, PMID: 24230945

[ref46] Vilar-Compte M. Vargas-Bustamante A. Lubben J. (2018). Validation study of the abbreviated version of the Lubben social network scale Spanish translation among Mexican and Mexican-American older adults. J. Cross Cult. Gerontol. 33, 83–99. 10.1007/s10823-017-9341-5, PMID: 29340902

[ref47] Wehrle K. Klehe U. -C. Kira M. Zikic J. (2018). Can I come as I am? Refugees’ vocational identity threats, coping, and growth. J. Vocat. Behav. 105, 83–101. 10.1016/j.jvb.2017.10.010

[ref48] Williams D. R. Yu Y. Jackson J. S. Anderson N. B. (1997). Racial differences in physical and mental health: socio-economic status, stress and discrimination. J. Health Psychol. 2, 335–351. 10.1177/135910539700200305 22013026

[ref49] Yamauchi F. Tanabe S. (2008). Nonmarket networks among migrants: evidence from metropolitan Bangkok, Thailand. J. Popul. Econ. 21, 649–664. 10.1007/s00148-006-0072-0

[ref50] Zikic J. (2015). Skilled migrants’ career capital as a source of competitive advantage: implications for strategic HRM. Int. J. Hum. Resour. Manag. 26, 1360–1381. 10.1080/09585192.2014.981199

[ref51] Zikic J. Bonache J. Cerdin J. L. (2010). Crossing national boundaries: a typology of qualified immigrants’ career orientations. J. Organ. Behav. 31, 667–686. 10.1002/job.705

[ref52] Zikic J. Richardson J. (2016). What happens when you can’t be who you are: professional identity at the institutional periphery. Hum. Relat. 69, 139–168. 10.1177/0018726715580865

